# Larval food plants of Australian Larentiinae (Lepidoptera: Geometridae) - a review of available data

**DOI:** 10.3897/BDJ.4.e7938

**Published:** 2016-03-21

**Authors:** Olga Schmidt

**Affiliations:** ‡ Zoologische Staatssammlung München, Munich, Germany

**Keywords:** Australasia, Australia, checklist, host plants, geometrid moths, larentiine moths

## Abstract

**Background:**

In Australia, the subfamily Larentiinae (Lepidoptera: Geometridae) comprises over 45 genera with about 270 species described so far. However, life histories of the Australian larentiine moths have barely been studied.

**New information:**

The current paper presents a list of larval food plants of 51 Australian larentiine species based on literature references, data from specimen labels and own observations. Some Australian habitats are shown. Possible relationships among the taxa based on food preference of the larvae are discussed. Additionally, a list of Australasian larentiine species from the genera occurring in Australia and their food plants is presented.

## Introduction

The immature stages and biology of the Australian Larentiinae (Lepidoptera, Geometridae) have received little attention in the past and our knowledge of host plant affiliations of the Australian species is remaining scarce. Hudson (1898) was one of the first researchers who discussed food plants of New Zealand larentiine larvae. Turner (1904), Common (1966) and Common (1990) presented some details of biology and listed a few food plants of Australian Larentiinae. McFarland (1979) published an annotated list of food plants of 280 Australian geometrid moths, including 16 larentiine species, whereby four species were identified to genus. He also succeeded to rear a large number of south Australian geometrid moths and completed 72 life history studies, but only four larentiine species were included (McFarland 1988). McQuillan (1986), McQuillan et al. (1998), McQuillan (1999) and McQuillan (2004) has been studying some aspects of biology, ecology and conservation of Australian moths focusing on the Tasmanian species. Holloway (1997) presented data on food plants of Indo-Australian Larentiinae. Some data on the larvae and food plants of the species *Anachloris* Meyrick, *Chaetolopha* Warren, *Scotocyma* Turner and *Visiana* Swinhoe are given in the reviews of these genera (Schmidt 2001, Schmidt 2002, Schmidt 2005, Schmidt 2006b, Schmidt 2007, Schmidt 2013.) Descriptions of larvae and pupae are incomplete or absent. Some observations on the eggs of Australian moths have been published by McFarland (1973). The first comprehensive review of the southern Australian geometrid eggs, including 18 larentiine species was completed by Young (2006), who also reared Tasmanian larvae of Geometridae, including several Larentiinae. Craw (1986) briefly described and illustrated a few New Zealand larentiine larvae. Totally, more than 270 larentiine species referred to about 45 genera are currently described from Australia. However, life histories of the vast majority of Australian larentiine moths remain unstudied.

## Materials and methods

The present report is based on literature references and personal observations. The following material has been used: *Anachloris uncinata* (Guenée) (Western Australia, Bremer Bay), *"Chloroclystis" approximata* (Walker) (New South Wales, Barren Grounds), *"Chloroclystis" insigillata* (Walker) (Queensland, Brisbane), *Epicyme rubropunctaria* (Doubleday) (New South Wales, Monga State Forest), *Epyaxa sodaliata* (Walker) (Queensland, Severnlea), *Gymnoscelis lophopus* Turner (Queensland, Brisbane), *Phrissogonus laticostata* (Walker) (Queensland, Brisbane), *Scotocyma albinotata* (Walker) (Queensland, Bunya Mountains), *Visiana brujata* (Guenée) (Queensland, Lamington National Park), *V. incertata* (Walker) (Queensland, Bunya Mountains). Additionally, data were taken from labels of specimens deposited in the Australian Na­tional Insect Collection, CSIRO, Ecosystem Sciences, Canberra (ANIC).

Taxonomic affiliation for several species is questionable therefore several names are cited in quotation marks. Tribal association is only cited for the first member of the tribe in the section “Nomenclature”. In the section “Notes” the source of data on the food plants is presented. A list of Australian species of Larentiinae and their larval food plants is available under “Supplementary Materials” (see Suppl. material 1). A list of Australasian larentiine species from the genera occurring in Australia and their food plants is also presented (see Suppl. material 2).

## Checklists

### List of the Australian Larentiinae (Geometridae) and their food plants

#### Epicyme rubropunctaria

(Doubleday, 1843)

Epicyme rubropunctaria Tribe Asthenini

##### Ecological interactions

###### Feeds on

*Geranium* sp. (Geraniaceae)

##### Notes

Roberts 1979. However, a newly hatched larva refused to feed on flowers and leaves of *Geranium* sp. (Schmidt, unpubl. data).

Fig. 1.

**Figure 2.**
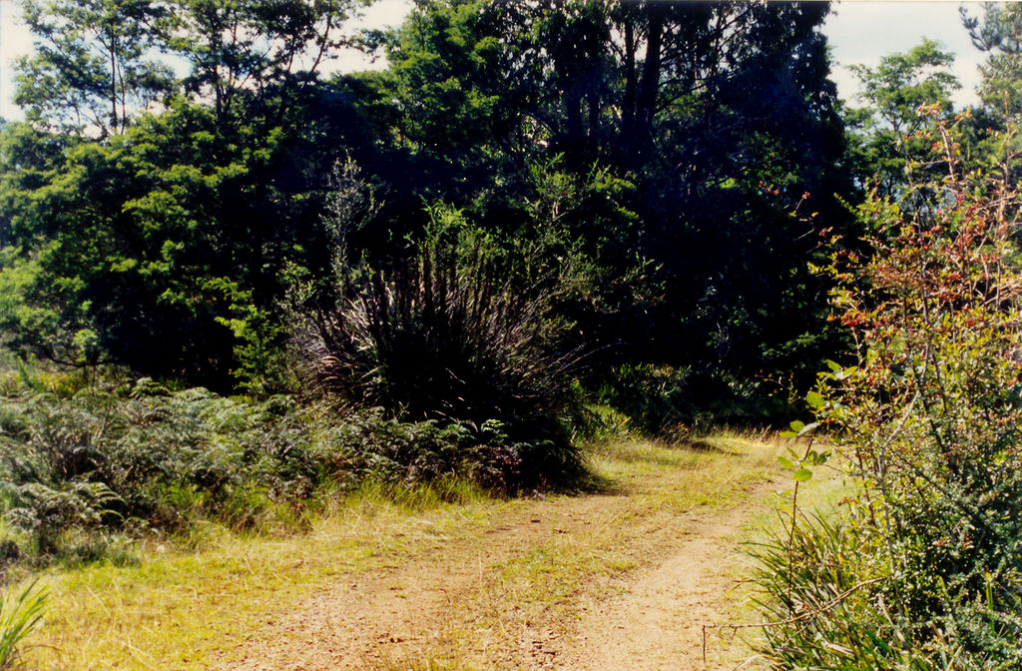
Habitat of *Epicyme rubropunctaria*, New South Wales, Monga State Forest

Habitat of *E. rubropunctaria* is presented on Fig. 2.

#### Epicyme rubropunctaria

(Doubleday, 1843)

##### Ecological interactions

###### Feeds on

*Haloragis alata* (Haloragaceae)

##### Notes

Hudson 1898.

#### Epicyme rubropunctaria

(Doubleday, 1843)

##### Ecological interactions

###### Feeds on

*Haloragis glauca* (Haloragaceae)

##### Notes

S. Williams, pers. comm., in: Marriott 2011.

#### Epicyme rubropunctaria

(Doubleday, 1843)

##### Ecological interactions

###### Feeds on

*Haloragis heterophylla* (Haloragaceae)

##### Notes

McFarland 1979.

#### Poecilasthena balioloma

(Turner, 1907)

##### Ecological interactions

###### Feeds on

*Leptospermum myrtifolium* (Myrtaceae)

##### Notes

McFarland 1979. Larvae of a New Zealand species *P. schistaria* (Walker, 1861) feed on *Leptospermum* sp. (Myrtaceae) (Hudson 1898).

#### Poecilasthena ischnophrica

Turner, 1941

##### Ecological interactions

###### Feeds on

*Leptospermum myrtifolium* (Myrtaceae)

##### Notes

McFarland 1979.

#### Poecilasthena ischnophrica

Turner, 1941

##### Ecological interactions

###### Feeds on

*Leptospermum myrsinoides* (Myrtaceae)

##### Notes

McFarland 1979.

#### Poecilasthena pulchraria

(Doubleday, 1843)

##### Ecological interactions

###### Feeds on

*Macropiper excelsum* (Piperaceae)

##### Notes

Hudson 1898.

#### Poecilasthena pulchraria

(Doubleday, 1843)

##### Ecological interactions

###### Feeds on

Monotoca? scoparia (Epacridaceae)

##### Notes

McQuillan 1986.

#### Poecilasthena pulchraria

(Doubleday, 1843)

##### Ecological interactions

###### Feeds on

*Monotoca glauca* (Epacridaceae)

##### Notes

C. Byrne, pers. comm., 2008.

#### Poecilasthena pulchraria

(Doubleday, 1843)

##### Ecological interactions

###### Feeds on

*Epacris* sp. (Epacridaceae)

##### Notes

McQuillan 1986.

#### Poecilasthena pulchraria

(Doubleday, 1843)

##### Ecological interactions

###### Feeds on

*Leucopogon juniperinus* (Epacridaceae)

##### Notes

McQuillan 1986.

#### Poecilasthena pulchraria

(Doubleday, 1843)

##### Ecological interactions

###### Feeds on

*Leptospermum scoparium* (Myrtaceae)

##### Notes

C. Byrne, pers. comm., 2008.

#### Poecilasthena pulchraria

(Doubleday, 1843)

##### Ecological interactions

###### Feeds on

*Astroloma humifusum* (Ericaceae)

##### Notes

McFarland 1979, McFarland 1988. Captured larvae were reared.

#### Poecilasthena pulchraria

(Doubleday, 1843)

##### Ecological interactions

###### Feeds on

*Brachyloma* sp. (Ericaceae)

##### Notes

Scoble 1999.

#### Poecilasthena xylocyma

(Meyrick, 1891)

##### Ecological interactions

###### Feeds on

*Leptospermum scoparium* (Myrtaceae)

##### Notes

ANIC label, C. Byrne, pers. comm., 2008.

#### Bosara minima

(Warren, 1897)

Bosara minima Tribe Eupitheciini

##### Ecological interactions

###### Feeds on

*Glochidion ferdinandi* (Euphorbiaceae)

##### Notes

Turner 1904. The plant species is described as *Phyllanthus ferdinandi*. An Indian larentiine species, *Bosara emarginaria* (Hampson, 1893) is known to feed on *Breynia* sp. (Euphorbiaceae) (P. Bell, pers. comm., in: Holloway 1997). The species *Bosara minima* was associated with the genera *Chloroclystis* Hübner and *Gymnoscelis* Mabille. The synonymy with *B. refusaria* Walker needs to be checked (see Holloway 1997).

#### "Chloroclystis" approximata

(Walker, 1869)

##### Ecological interactions

###### Feeds on

*Malus domestica* (Rosaceae)

##### Notes

Common 1990. Larvae occasionally damage the young fruits of apples.

Fig. 3.

**Figure 4.**
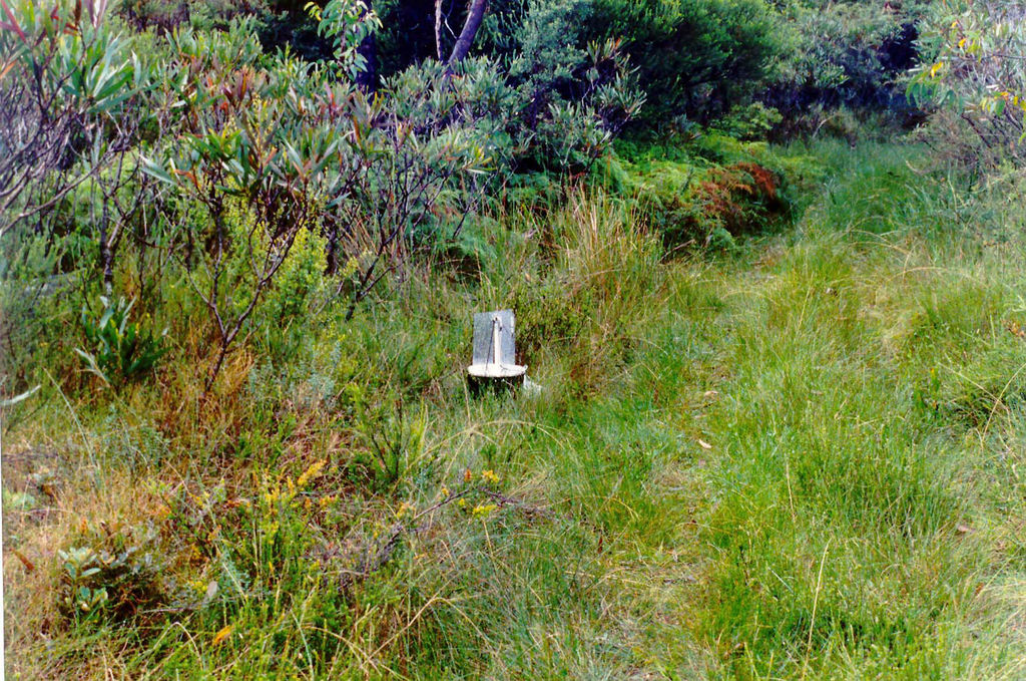
Habitat of *"Chloroclystis" approximata*, New South Wales, Barren Grounds

Habitat of *C. approximata* is presented on Fig. 4.

#### "Chloroclystis" approximata

(Walker, 1869)

##### Ecological interactions

###### Feeds on

*Prunus avium* (Rosaceae)

##### Notes

Common 1990. Larvae occasionally damage the young fruits of cherries.

#### "Chloroclystis" approximata

(Walker, 1869)

##### Ecological interactions

###### Feeds on

*Acacia terminalis* (Fabaceae)

##### Notes

Turner 1904, McQuillan 1986, Common 1990, Schmidt, unpubl. data. Larvae usually feed on the flowers of *Acacia* sp. The foodplant is known as *Acacia botrycephala*.

#### "Chloroclystis" catastreptes

(Meyrick, 1891)

##### Ecological interactions

###### Feeds on

*Bertya mitchellii* (Euphorbiaceae)

##### Notes

McFarland 1979. Larvae feed on flowers and flower buds of various unrelated plants.

#### "Chloroclystis" catastreptes

(Meyrick, 1891)

##### Ecological interactions

###### Feeds on

*Acacia* sp. (Fabaceae)

##### Notes

McFarland 1979, McQuillan 1986. Larvae feed on flowers and flower buds of various unrelated plants.

#### "Chloroclystis" catastreptes

(Meyrick, 1891)

##### Ecological interactions

###### Feeds on

*Clematis microphylla* (Ranunculaceae)

##### Notes

McFarland 1979. Larvae feed on flowers and flower buds of various unrelated plants.

#### "Chloroclystis" catastreptes

(Meyrick, 1891)

##### Ecological interactions

###### Feeds on

*Solidago* sp. (Asteraceae)

##### Notes

McFarland 1979, McQuillan 1986. Larvae feed on flowers and flower buds of various unrelated plants.

#### "Chloroclystis" filata

(Guenée, 1858)

##### Ecological interactions

###### Feeds on

*Pultenaea largiflorens var. latifolia* (Fabaceae)

##### Notes

McFarland 1979. Larvae feed on leaves and buds of the foodplant.

#### "Chloroclystis" filata

(Guenée, 1858)

##### Ecological interactions

###### Feeds on

*Hebe* sp. (Plantaginaceae)

##### Notes

White 1991.

#### "Chloroclystis" insigillata

(Walker, 1863)

##### Ecological interactions

###### Feeds on

*Macadamia* sp. (Proteaceae)

##### Notes

Common 1990. Larvae attack the flowers of *Macadamia* sp.

#### "Chloroclystis" insigillata

(Walker, 1863)

##### Ecological interactions

###### Feeds on

*Acacia* sp. (Fabaceae)

##### Notes

Common 1990, Schmidt, unpubl. data.

#### "Chloroclystis" insigillata

(Walker, 1863)

##### Ecological interactions

###### Feeds on

*Bertya* sp. (Euphorbiaceae)

##### Notes

Common 1990.

#### "Chloroclystis" insigillata

(Walker, 1863)

##### Ecological interactions

###### Feeds on

*Clematis* sp. (Ranunculaceae)

##### Notes

Common 1990, Schmidt, unpubl. data.

#### "Chloroclystis" insigillata

(Walker, 1863)

##### Ecological interactions

###### Feeds on

*Solidago* sp. (Asteraceae)

##### Notes

Common 1990.

#### Chloroclystis s.l. sp.

##### Ecological interactions

###### Feeds on

Scrophulariaceae

##### Notes

McQuillan 1986.

#### Collix ghosha

(Walker, 1863)

##### Ecological interactions

###### Feeds on

*Ardisia* sp. (Primulaceae)

##### Notes

P. Bell, pers. comm., in: Holloway 1997, Tominaga 1998. Bell describes biology of *Collix ghosha*. The foodplant *Ardisia* sp. was in the former Myrsinaceae. A south-east Asian species *C. griseipalpis* Wileman, 1916 has been reared from *Allophylus* sp. (Sapindaceae). A subspecies *C. g. phaeochiton* Prout, 1932 has been reared from *Ardisia* sp. and *Trigonostemon* sp. (Euphorbiaceae) (Prout 1932).

#### Collix ghosha

(Walker, 1863)

##### Ecological interactions

###### Feeds on

*Embelia* sp. (Primulaceae)

##### Notes

P. Bell, pers. comm., in: Holloway 1997, Tominaga 1998. Bell describes biology of *Collix ghosha*. The foodplant *Embelia* sp. was in the former Myrsinaceae.

#### Gymnoscelis delocyma

Turner, 1904

##### Ecological interactions

###### Feeds on

*Scyphiphora hydrophyllaceae* (Rubiaceae)

##### Notes

F.P. Dodd, pers. comm., in: Turner 1904. The larvae of the Malaysian species *Gymnoscelis pseudotibialis* Holloway, 1997 apparently feed on *Hevea* sp. (Euphorbiaceae) and *Mangifera* sp. (Anacardiaceae) (Yunus & Ho 1980, in: Holloway 1997).

#### Gymnoscelis derogata

(Walker, 1866)

##### Ecological interactions

###### Feeds on

*Macadamia* sp. (Proteaceae)

##### Notes

Zhang 1994. The species is known as *Gymnoscelis subrufata* Warren, 1898.

#### Gymnoscelis lophopus

Turner, 1904

##### Ecological interactions

###### Feeds on

*Acacia aulacocarpa* (Fabaceae)

##### Notes

Turner 1904, Common 1990. Larvae feed on the flowers of the foodplant.

Fig. 5.

**Figure 6.**
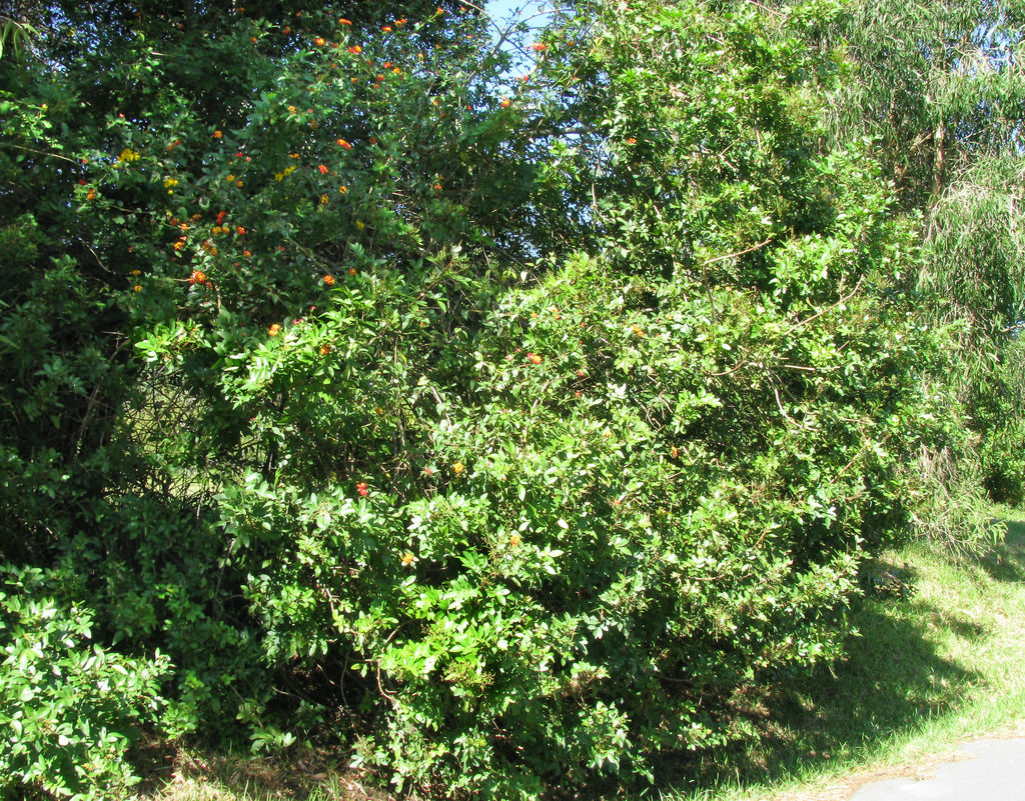
Habitat of *Gymnoscelis lophopus*, Queensland, Brisbane

Habitat of *Gymnoscelis lophopus* is presented on Fig. 6.

#### Gymnoscelis lophopus

Turner, 1904

##### Ecological interactions

###### Feeds on

*Lantana camara* (Verbenaceae)

##### Notes

Schmidt, unpubl. data. Larvae feed on the flowers of the foodplant.

#### Gymnoscelis lophopus

Turner, 1904

##### Ecological interactions

###### Feeds on

*Lantana* sp. (Verbenaceae)

##### Notes

Common 1990.

#### Gymnoscelis lophopus

Turner, 1904

##### Ecological interactions

###### Feeds on

*Macadamia* sp. (Proteaceae)

##### Notes

Common 1990. Larvae sometimes damage the flowers of *Macadamia* sp.

#### Gymnoscelis sp.

##### Ecological interactions

###### Feeds on

*Pittosporum venulosum* (Pittosporaceae)

##### Notes

D. Herbison-Evans, pers. comm., 2015.

#### Microdes oriochares

Turner, 1922

##### Ecological interactions

###### Feeds on

*Olearia ramulosa* (Asteraceae)

##### Notes

McFarland 1979. Larvae feed on leaves of the foodplant.

#### Microdes squamulata

Guenée, 1858

##### Ecological interactions

###### Feeds on

*Acacia baileyana* (Fabaceae)

##### Notes

McFarland 1979.

#### Microdes squamulata

Guenée, 1858

##### Ecological interactions

###### Feeds on

*Acacia buxifolia* (Fabaceae)

##### Notes

McFarland 1979.

#### Microdes squamulata

Guenée, 1858

##### Ecological interactions

###### Feeds on

*Acacia dealbata* (Fabaceae)

##### Notes

McFarland 1979.

#### Microdes squamulata

Guenée, 1858

##### Ecological interactions

###### Feeds on

*Acacia decurrens* (Fabaceae)

##### Notes

Turner 1904.

#### Microdes squamulata

Guenée, 1858

##### Ecological interactions

###### Feeds on

*Acacia mearnsii* (Fabaceae)

##### Notes

McFarland 1979.

#### Microdes villosata

Guenée, 1858

##### Ecological interactions

###### Feeds on

*Acacia* sp. (Fabaceae)

##### Notes

McQuillan 1999.

#### Pasiphila testulata

(Guenée, 1858)

##### Ecological interactions

###### Feeds on

*Malus domestica* (Rosaceae)

##### Notes

Common 1990. Larvae occasionally damage the young fruits of apples. Three Europaean species, *P. chloerata* (Mabille, 1870), *P. debiliata* (Hübner, 1817) and *P. rectangulata* (Linnaeus, 1758) feed on *Prunus* spp. (Rosaceae), *Vaccinium* spp. (Ericaceae) and *Malus* sp., *Pyrus* sp., *Prunus* spp., *Crataegus* sp. and *Amelanchier* sp. (Rosaceae) (Mironov 2003). A New Zealand species *P. urticae* (Hudson, 1939) feed on *Urtica ferox* (Urticaceae).

#### Pasiphila testulata

(Guenée, 1858)

##### Ecological interactions

###### Feeds on

*Prunus avium* (Rosaceae)

##### Notes

Common 1990. Larvae occasionally damage the young fruits of cherries. *P. testulata* is known as *Chloroclystis testulata* (Guenée).

#### Pasiphila testulata

(Guenée, 1858)

##### Ecological interactions

###### Feeds on

*Acacia terminalis* (Fabaceae)

##### Notes

Common 1990, Schmidt, unpubl. data, C. Byrne, pers. comm., 2008. Larvae usually feed on the flowers of *Acacia* sp. The foodplant is known as *Acacia botrycephala*.

#### Phrissogonus laticostata

(Walker, 1863)

##### Ecological interactions

###### Feeds on

*Acacia* sp. (Fabaceae)

##### Notes

Common 1990. Larvae usually feed on the flower buds and flowers of *Acacia*.

#### Phrissogonus laticostata

(Walker, 1863)

##### Ecological interactions

###### Feeds on

*Clematis* sp. (Ranunculaceae)

##### Notes

Common 1990. Larvae can damage the foliage of the foodplant.

#### Phrissogonus laticostata

(Walker, 1863)

##### Ecological interactions

###### Feeds on

*Cosmos* sp. (Asteraceae)

##### Notes

Schmidt, unpubl. data. Larvae readily accepted flowers of *Cosmos* sp. from a garden in Brisbane.

#### Phrissogonus laticostata

(Walker, 1863)

##### Ecological interactions

###### Feeds on

*Helianthus annuus* (Asteraceae)

##### Notes

Common 1990. Larvae can damage the foliage of the foodplant.

#### Phrissogonus laticostata

(Walker, 1863)

##### Ecological interactions

###### Feeds on

*Hypericum* sp. (Hypericaceae)

##### Notes

Common 1990. Larvae can damage the foliage of the foodplant.

#### Phrissogonus laticostata

(Walker, 1863)

##### Ecological interactions

###### Feeds on

*Malus domestica* (Rosaceae)

##### Notes

Common 1990. Larvae can damage the foliage of the foodplant.

#### Phrissogonus laticostata

(Walker, 1863)

##### Ecological interactions

###### Feeds on

*Medicago sativa* (Fabaceae)

##### Notes

Zhang 1994.

#### Phrissogonus laticostata

(Walker, 1863)

##### Ecological interactions

###### Feeds on

*Prunus avium* (Rosaceae)

##### Notes

Zhang 1994.

#### Phrissogonus laticostata

(Walker, 1863)

##### Ecological interactions

###### Feeds on

*Prunus cerasus* (Rosaceae)

##### Notes

Zhang 1994.

#### Phrissogonus laticostata

(Walker, 1863)

##### Ecological interactions

###### Feeds on

*Rosa odorata* (Rosaceae)

##### Notes

D. Herbison-Evans, pers. comm., 2015. Captured larvae readily accepted the flower petals from *Rosa* sp.

#### Symmimetis sp.

##### Ecological interactions

###### Feeds on

*Aglaia* sp. (Meliaceae)

##### Notes

Holloway 1997.

#### Sauris cirrhigera

(Warren, 1897)

Sauris cirrhigera Tribe Trichopterygini

##### Ecological interactions

###### Feeds on

*Cinnamomum* sp. (Lauraceae)

##### Notes

Dugdale 1980. One specimen of the Indo-Pacific species *Sauris eupitheciata* (Snellen, 1881) was reared from the folliage of *Loranthus* sp. (Loranthaceae) (Holloway 1997), of *Sauris hirudinata* Guenée, 1858 from *Alseodaphne* sp. (Lauraceae) and *Lagerstroemia* sp. (Lythraceae) (P. Bell, pers. comm., in: Holloway 1997), of *Sauris interruptata* (Moore, 1888) on *Cinnamomum* sp. (Lauraceae) Holloway 1997), and of one species occurring on Niue Island on *Ficus prolixa* (Moraceae) (Dugdale 1980).

#### Sauris commoni

Dugdale, 1980

##### Ecological interactions

###### Feeds on

*Exocarpos latifolius* (Santalaceae)

##### Notes

ANIC label. One larva was beaten from *Exocarpos latifolia*.

#### Sauris malaca

(Meyrick, 1891)

##### Ecological interactions

###### Feeds on

*Litchi chinensis* (Sapindaceae)

##### Notes

Dugdale 1980, Common 1990. Larvae have been reported feeding on the young foliage of *Litchi* sp.

#### Sauris malaca

(Meyrick, 1891)

##### Ecological interactions

###### Feeds on

*Toona ciliata* (Meliaceae)

##### Notes

Dugdale 1980, Common 1990. Larvae have been reported feeding on the young foliage of *Toona* sp. The foodplant is known as *Toona australis*.

#### Tympanota perophora

(Turner, 1922)

##### Ecological interactions

###### Feeds on

*Podocarpus lawrencei* (Podocarpaceae)

##### Notes

ANIC label, Dugdale 1980. The species has been reared by I.F.B. Common (ANIC).

#### Acodia sp.

Acodia sp. Tribe Xanthorhoini

##### Ecological interactions

###### Feeds on

*Coprosma* sp. (Rubiaceae)

##### Notes

McQuillan 1999, McQuillan 2004.

#### Austrocidaria sp.

##### Ecological interactions

###### Feeds on

*Coprosma* sp. (Rubiaceae)

##### Notes

Hudson 1898, Dugdale 1964, Dugdale 1988, McQuillan 1999, McQuillan 2004. One New Zealand species feeds on *Myrsine* sp. and *Rapanea crassifolia* (Myrsinaceae) (Dugdale 1971).

#### Chrysolarentia decisaria

(Walker, 1863)

##### Ecological interactions

###### Feeds on

*Pelargonium rodneyanum* (Geraniaceae)

##### Notes

McFarland 1979, McFarland 1988, C. Byrne, pers. comm., 2008.

#### Chrysolarentia decisaria

(Walker, 1863)

##### Ecological interactions

###### Feeds on

*Ranunculus prasinus* (Ranunculaceae)

##### Notes

McFarland 1979, McFarland 1988, C. Byrne, pers. comm., 2008.

#### Chrysolarentia insulsata

(Guenée, 1858)

##### Ecological interactions

###### Feeds on

*Plantago lanceolata* (Plantaginaceae)

##### Notes

McFarland 1979, McFarland 1988.

#### Chrysolarentia lucidulata

(Walker, 1963)

##### Ecological interactions

###### Feeds on

*Plantago lanceolata* (Plantaginaceae)

##### Notes

McFarland 1979, McFarland 1988.

#### Chrysolarentia vicissata

(Guenée, 1858)

##### Ecological interactions

###### Feeds on

*Hibbertia* sp. (Dilleniaceae)

##### Notes

McFarland 1979 McFarland 1988.

#### Chrysolarentia vicissata

(Guenée, 1858)

##### Ecological interactions

###### Feeds on

*Lythrum* sp. (Lythraceae)

##### Notes

McFarland 1979, McFarland 1988.

#### Chrysolarentia vicissata

(Guenée, 1858)

##### Ecological interactions

###### Feeds on

*Malva* sp. (Malvaceae)

##### Notes

McFarland 1979, McFarland 1988.

#### Chrysolarentia vicissata

(Guenée, 1858)

##### Ecological interactions

###### Feeds on

*Mentha* sp. (Lamiaceae)

##### Notes

McFarland 1979, McFarland 1988.

#### Chrysolarentia vicissata

(Guenée, 1858)

##### Ecological interactions

###### Feeds on

*Polygonum* sp. (Polygonaceae)

##### Notes

McFarland 1979, McFarland 1988.

#### Chrysolarentia vicissata

(Guenée, 1858)

##### Ecological interactions

###### Feeds on

*Centaurium* sp. (Gentianaceae)

##### Notes

McFarland 1979, McFarland 1988. Larvae were feeding on introduced weeds in capture.

#### Chrysolarentia vicissata

(Guenée, 1858)

##### Ecological interactions

###### Feeds on

*Chenopodium* sp. (Chenopodiaceae)

##### Notes

McFarland 1979, McFarland 1988. Larvae were feeding on introduced weeds in capture.

#### Chrysolarentia vicissata

(Guenée, 1858)

##### Ecological interactions

###### Feeds on

*Medicago* sp. (Fabaceae)

##### Notes

McFarland 1979, McFarland 1988. Larvae were feeding on introduced weeds in capture.

#### Chrysolarentia vicissata

(Guenée, 1858)

##### Ecological interactions

###### Feeds on

*Plantago* sp. (Plantaginaceae)

##### Notes

McFarland 1979, McFarland 1988. Larvae were feeding on introduced weeds in capture.

#### Chrysolarentia vicissata

(Guenée, 1858)

##### Ecological interactions

###### Feeds on

*Solidago* sp. (Asteraceae)

##### Notes

McFarland 1979, McFarland 1988. Larvae were feeding on introduced weeds in capture.

#### Chrysolarentia vicissata

(Guenée, 1858)

##### Ecological interactions

###### Feeds on

*Stellaria* sp. (Caryophyllaceae)

##### Notes

McFarland 1979, McFarland 1988. Larvae were feeding on introduced weeds in capture.

#### Epyaxa sodaliata

(Walker, 1963)

##### Ecological interactions

###### Feeds on

*Anagallis arvensis* (Primulaceae)

##### Notes

McFarland 1979. Larvae readily accepted leaves and buds of *Anagallis arvensis* but refused to feed on *Plantago*. A New Zealand species *E. rosearia* (Doubleday, 1843) feeds on *Nasturtium officinale* (Brassicaceae) (Hudson 1898).

Fig. 7.

**Figure 8.**
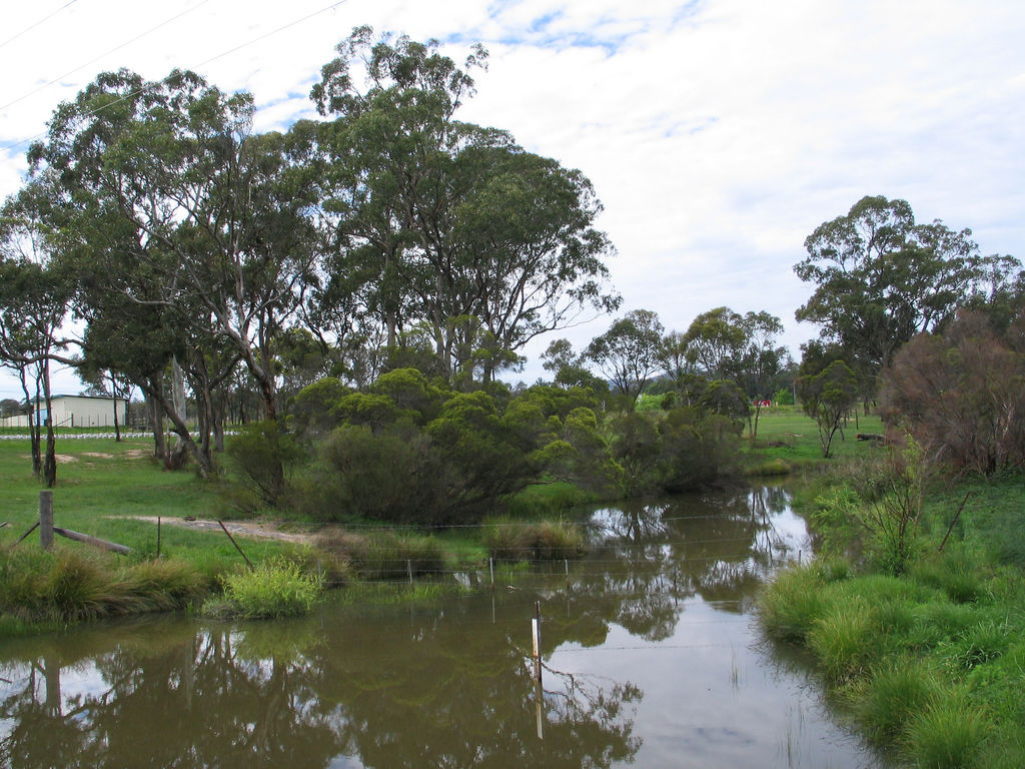
*Habitat of Epyaxa sodaliata*, Queensland, Severnlea

Habitat of *Epyaxa sodaliata* is presented on Fig. 8.

#### Epyaxa sodaliata

(Walker, 1963)

##### Ecological interactions

###### Feeds on

*Primula* sp. (Primulaceae)

##### Notes

Schmidt, unpubl. data. Larvae were feeding on *Primula* sp. from a garden in Brisbane.

#### Epyaxa sodaliata

(Walker, 1963)

##### Ecological interactions

###### Feeds on

*Myosotis arvensis* (Boraginaceae)

##### Notes

D. Herbison-Evans, pers. comm., 2015.

#### Epyaxa subidaria

(Guenée, 1858)

##### Ecological interactions

###### Feeds on

*Medicago polymorpha* var. vulgaris (Fabaceae)

##### Notes

McFarland (1979), Schmidt, unpubl. data. Captured larvae were reared.

#### Epyaxa subidaria

(Guenée, 1858)

##### Ecological interactions

###### Feeds on

*Hydrocotyle sibthorpioides* (Araliaceae)

##### Notes

McQuillan (1999). Captured larvae were reared to the final instar.

#### Epyaxa subidaria

(Guenée, 1858)

##### Ecological interactions

###### Feeds on

*Plantago lanceolata* (Plantaginaceae)

##### Notes

McQuillan (2004). One New Zealand *Epyaxa* species is known to feed on *Rumex* sp. (Polygonaceae) and *Tropaeolum majus* (Tropaeolaceae) (White 1991).

#### Scotocyma albinotata

(Walker, 1866)

##### Ecological interactions

###### Feeds on

*Coprosma repens* (Rubiaceae)

##### Notes

Schmidt (2003), Schmidt (2005), Schmidt (2006a), Schmidt (2007).

Fig. 9.

**Figure 10.**
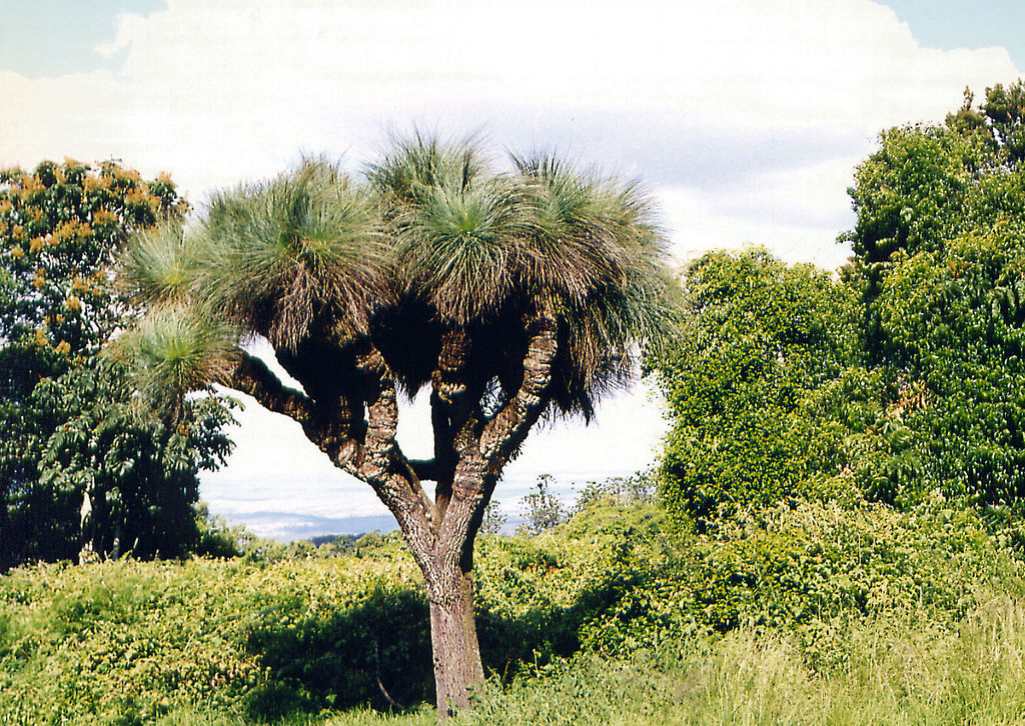
Habitat of *Scotocyma albinotata*, Queensland, Bunya Mountains

Habitat of *Scotocyma albinotata* is presented on Fig. 10.

#### Xanthorhoe vacuaria

(Guenée, 1858)

##### Ecological interactions

###### Feeds on

*Medicago polymorpha* var. vulgaris (Fabaceae)

##### Notes

McFarland (1979), McFarland (1988). Captured larvae were reared. Malaysian *Xanthorhoe liwagu* Holloway, 1997 were feeding on *Brassica* sp. (Cruciferae) and *Mentha* sp. (Labiatae) (Yunus and Ho 1980, Singh 1953, in: Holloway 1997).

#### Anachloris subochraria

(Doubleday)

Anachloris subochraria Unplaced to tribe

##### Ecological interactions

###### Feeds on

Epilobium? ciliatum (Onagraceae)

##### Notes

S. Williams, unpubl. data.

#### Anachloris tofocolorata

Schmidt, 2001

##### Ecological interactions

###### Feeds on

*Hibbertia virgata* (Dilleniaceae)

##### Notes

McFarland (1979), Schmidt (2001).

#### Anachloris uncinata

(Guenée)

##### Ecological interactions

###### Feeds on

*Hibbertia obtusifolia* (Dilleniaceae)

##### Notes

Common (1966), McFarland (1979), Schmidt (2001).

Fig. 11.

**Figure 12.**
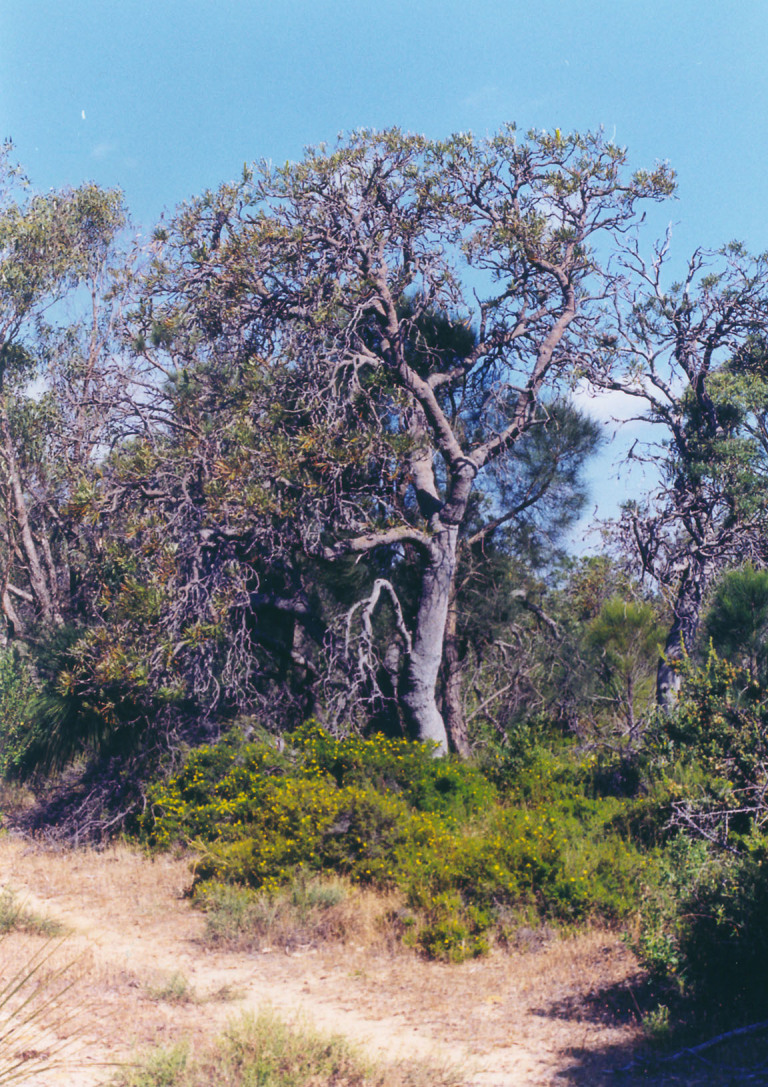
Habitat of *Anachloris uncinata*, Western Australia, Stirling Range

Habitat of *A. uncinata* is presented on Fig. 12.

#### Anachloris uncinata

(Guenée)

##### Ecological interactions

###### Feeds on

*Hibbertia riparia* (Dilleniaceae)

##### Notes

S. Williams, unpubl. data.

#### Anachloris uncinata

(Guenée, 1858)

##### Ecological interactions

###### Feeds on

*Hibbertia stricta* (Dilleniaceae)

##### Notes

McFarland (1979), Schmidt (2001).

#### Chaetolopha emporias

(Turner, 1904)

##### Ecological interactions

###### Feeds on

*Pteridium esculentum* (Polypodiaceae)

##### Notes

ANIC label, Schmidt (2002). In ANIC there is a specimen with a label written by I.F.B. Common, “Larvae eat bracken fern".

#### "Chrysolarentia" actinipha

(Lower, 1902)

##### Ecological interactions

###### Feeds on

*Medicago polymorpha* var. vulgaris (Fabaceae)

##### Notes

McFarland (1979), McFarland (1988).

#### "Chrysolarentia" leucophanes

(Meyrick, 1891)

##### Ecological interactions

###### Feeds on

*Leptospermum scoparium* (Myrtaceae)

##### Notes

C. Byrne, pers. comm., 2008.

#### "Chrysolarentia" leucophanes

(Meyrick, 1891)

##### Ecological interactions

###### Feeds on

*Melaleuca squamea* (Myrtaceae)

##### Notes

C. Byrne, pers. comm., 2008.

#### "Chrysolarentia" leucophanes

(Meyrick, 1891)

##### Ecological interactions

###### Feeds on

*Monotoca glauca* (Epacridaceae)

##### Notes

C. Byrne, pers. comm., 2008.

#### "Chrysolarentia" severata

(Guenée, 1858)

##### Ecological interactions

###### Feeds on

*Astroloma humifusum* (Ericaceae)

##### Notes

S. Williams, pers. comm., in: Marriott (2011).

#### "Chrysolarentia" severata

##### Ecological interactions

###### Feeds on

*Leptospermum scoparium* (Myrtaceae)

##### Notes

C. Byrne, pers. comm., 2016. The species is recorded as "Euphyia" nr. severata. The collection details are: Cape Bruny, Tasmania, 28/10/99, C. Byrne.

#### "Chrysolarentia" squamulata

(Warren, 1899)

##### Ecological interactions

###### Feeds on

*Olearia ramulosa* (Asteraceae)

##### Notes

McFarland (1979).

#### Heterohasta conglobata

(Walker, 1963)

##### Ecological interactions

###### Feeds on

*Hibbertia scandens* (Dilleniaceae)

##### Notes

ANIC label. Larvae feed on leaves and shoots of *Hibbertia scandens*.

#### Melitulias sp.

##### Ecological interactions

###### Feeds on

Fabaceae

##### Notes

McQuillan (1986).

#### Melitulias s.l. sp. undescribed

##### Ecological interactions

###### Feeds on

*Casuarina paludosa* var. robusta (Casuarinaceae)

##### Notes

McFarland (1988). The species cited as “*Horisme*” sp.? has been reared. The specimen apparently represents an undescribed species (Schmidt, unpubl. data).

#### Polyclysta hypogrammata

Guenée, 1858

##### Ecological interactions

###### Feeds on

*Ficus* sp. (Moraceae)

##### Notes

Turner (1904).

#### Visiana brujata

(Guenée, 1858)

##### Ecological interactions

###### Feeds on

*Urtica incisa* (Urticaceae)

##### Notes

ANIC label, Schmidt (2006b), Schmidt (2013). Larvae were reared from eggs.

#### Visiana incertata

(Walker, 1862)

##### Ecological interactions

###### Feeds on

*Urtica incisa* (Urticaceae)

##### Notes

Schmidt, unpubl. data. Larvae were reared from eggs.

Fig. 13.

**Figure 14.**
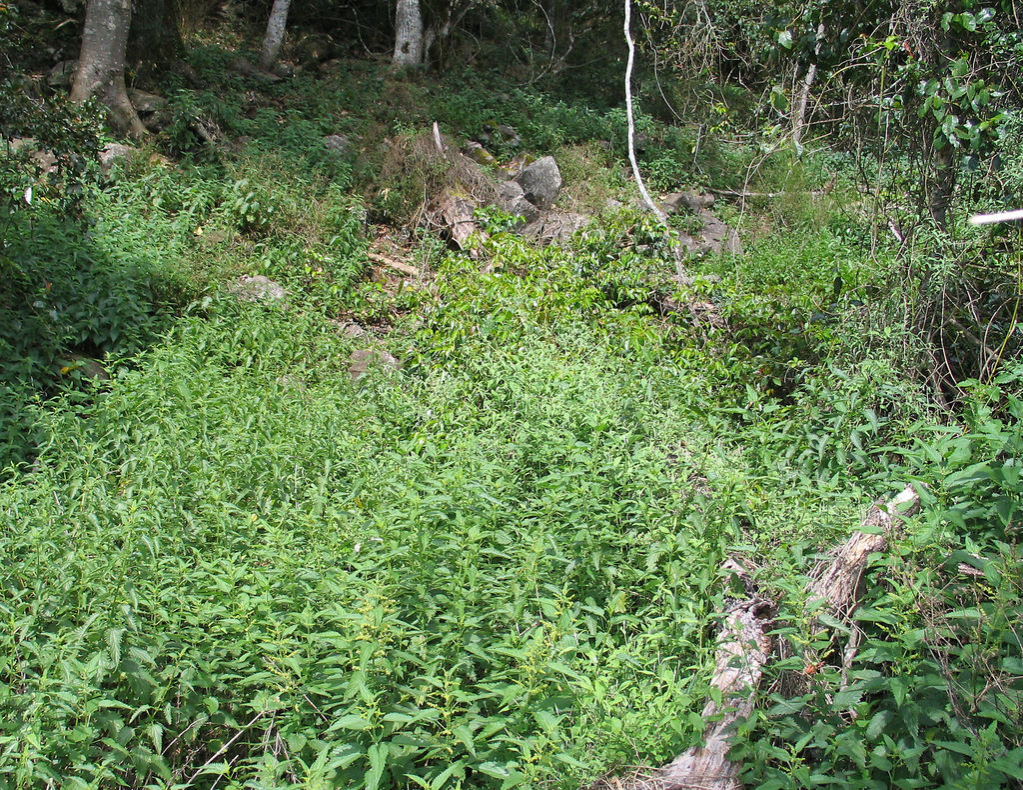
Habitat of *Visiana incertata*, Bunya Mountains

Habitat of *Visiana incertata* is presented on Fig. 14.

#### Visiana incertata

(Walker, 1862)

##### Ecological interactions

###### Feeds on

*Urtica dioica* (Urticaceae)

##### Notes

Schmidt, unpubl. data. Final instar larvae readily accepted the leaves of *Urtica dioica* (flowers and buds were not offered).

## Discussion

Larval food plants of 51 Australian larentiine species from the following tribes are presented, including Asthenini (5 species), Eupitheciini (17 species), Trichopterygini (4 species) and Xanthorhoini (10 species). Additionally, food plants of 15 species unplaced to tribe are listed. The larvae are recorded to feed on 36 plant families (Table 1). More than a half of plant species are native to Australia. Two species, namely *Lantana camara* (Verbenaceae) and *Acacia mearnsii* (Fabaceae) are recorded as invasive species.

*"Chloroclystis" approximata*, *"C." insigillata*, *Gymnoscelis lophopus*, *G. derogata*, *Pasiphila testulata*, *Phrissogonus laticostata* and *Sauris malaca* are known as minor pests of cultivated plants.

The food plants are recorded for about 20% of Australian species therefore conclusions about food preference are rather preliminary. Moreover, the larentiine larvae are often polyphagous, hence the assumptions that taxa are closely related based solely on food preference of the larvae should not be overestimated.

### Tribes Asthenini and Eupitheciini

Like in the Palaearctic region, larvae of Australian species of the tribe Eupitheciini are mostly polyphagous or oligophagous, tending to feed on flowers and buds of various plants. The tribes Eupitheciini and Asthenini are often considered closely related (*e.g.* Xue and Scoble 2002). Holloway (1997) treated the ‘asthenine’ genera in Eupitheciini, although he mentioned that *Poecilasthena* Warren, *Parasthena* Warren, *Eois* Hübner, *Polynesia* Swinhoe and *Pseudopolynesia* Holloway could be placed in Ashtenini. The present study revealed no evidence of concordance of the data on food preference of the larvae of these two tribes. The asthenine larvae mainly feed on leaves of native Epacridaceae, Ericaceae, Haloragaceae and Myrtaceae, with one species feeding on Piperaceae, whereas the larvae of Eupitheciini prefer feeding on the flowers and buds of Asteraceae, Euphorbiaceae, Fabaceae, Hypericaceae, Pittosporaceae, Plantaginaceae, Primulaceae, Proteaceae, Ranunculaceae, Rosaceae, Rubiaceae, Verbenaceae and occasionally on Meliaceae and Menispermaceae. The tribe Asthenini seems to be distinct from Eupitheciini, however, additional data need to be collected and analysed to clarify placement of several genera currently included in these tribes.

### Tribe Trichopterygini

Food plants are recognized for several Indo-Pacific and South American species of the genera occurring in Australia. Larvae of one Japanese species of *Episteira* Warren from the tribe Trichopterygini feed on foliage of trees or shrubs of *Podocarpus* sp. (Podocarpaceae) (Sugi, 1987, in: Holloway 1997), like Australian trichopterygine species of *Tympanota*. Generally, the Australian trichopterygines are associated with Lauraceae, Meliaceae, Santalaceae and Sapindaceae. In Europe, larvae of Trichopterygini are associated with trees and shrubs from the families Anacardiaceae, Cupressaceae, Salicaceae and Sapindaceae, with a few polyphagous species feeding on Aquifoliaceae, Araliaceae, Caprifoliaceae, Cornaceae, Ranunculaceae, Rhamnaceae and Rosaceae (see Hausmann and Viidalepp 2012). Most of the trichopterygine food plants belong to the Sapindales in both Europe and Australasia.

### Tribe Xanthorhoini

Like in the Palaearctic region, larvae of Australian xanthorhoines are polyphagous, feeding mainly on foliage of flowering plants and herbs. Most of the Australian larvae accepted Plantaginaceae, Fabaceae and Rubiaceae.

### Genera unplaced to tribes

Larvae of a New Zealand species *Aponotoreas dissimilis* (Philpott, 1914) accepted *Dracophyllum* sp. (Epacridaceae), whereas *A. synclinalis* (Hudson, 1903) was feeding on *Empodisma minus* (Restionaceae) (B. Patrick, pers. comm., in: Craw 1986). The genus *Aponotoreas* Craw is currently assigned to the tribe Hydriomenini (McQuillan and Edwards 1996) but does not share several morphological characters of the tribe and is in need of taxonomic study (Schmidt, unpubl. data). Epacridaceae is a food plant of several asthenine species and of *Chrysolarentia leucophanes* of which the tribal assignment is still unclear. Apart from *A. synclinalis*, no further larentiine larvae are known to feed on Restionaceae. In Europe, the larvae of *Hydriomena* spp. are known to feed on Betulaceae, Corylaceae, Ericaceae, Fagaceae and Salicaceae (see Hausmann and Viidalepp 2012). Regarding the larval food preference of *Aponotoreas*, there is no indication of a close affinity with Hydriomenini.

Larvae of an Indo-Pacific species *Eois grataria* (Walker, 1861) feed on *Mallotus* sp. (Euphorbiaceae) (Singh, 1953, in: Holloway 1997), while most of South American species of the genus readily accept *Piper* sp. (Piperaceae) (Strutzenberger and Fiedler 2011). *Eois* Hübner is not assigned to any tribe currently although it has been cited in Asthenini and Eupitheciini, or excluded from both tribes (Holloway 1997, Xue and Scoble 2002, Strutzenberger and Fiedler 2011, Viidalepp 2011). *Glochidion* sp., *Bertya* sp., *Trigonostemon* sp., *Hevea* sp. (Euphorbiaceae) are food plants of the Indo-Australian eupitheciine larvae, which would indicate an affinity of *Eois* with Eupitheciini. However, larvae of one asthenine species feed on Piperaceae, like *Poecilasthena pulchraria* (Doubleday, 1843) that is placed in Asthenini. Adult morphological characters indicate a close relationship of the Australasian *Eois* to Eupitheciini (Schmidt, unpubl. data).

## Supplementary Material

Supplementary material 1 List of the Australian Larentiinae (Geometridae) and their food plants Data type: food plants
File: oo_81963.xls O. Schmidt

Supplementary material 2 List of the Australasian Larentiinae (Geometridae) and their food plants Data type: food plants
File: oo_72989.xls O. Schmidt

## Figures and Tables

**Figure 1.**
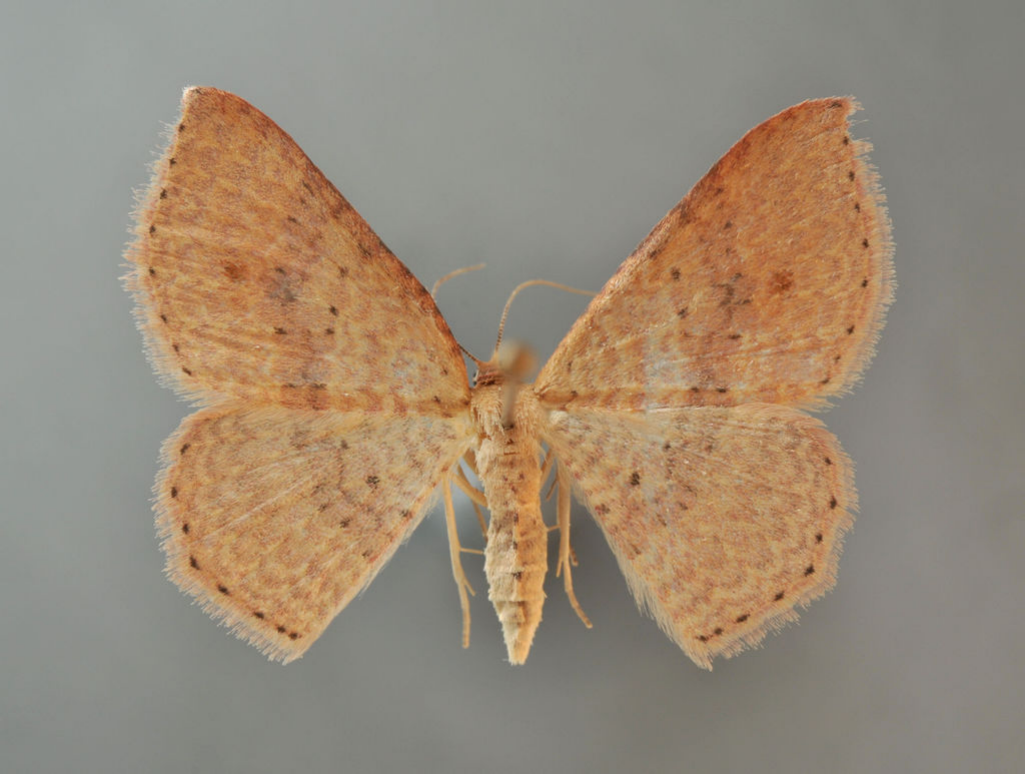
*Epicyme rubropunctaria*, female

**Figure 3.**
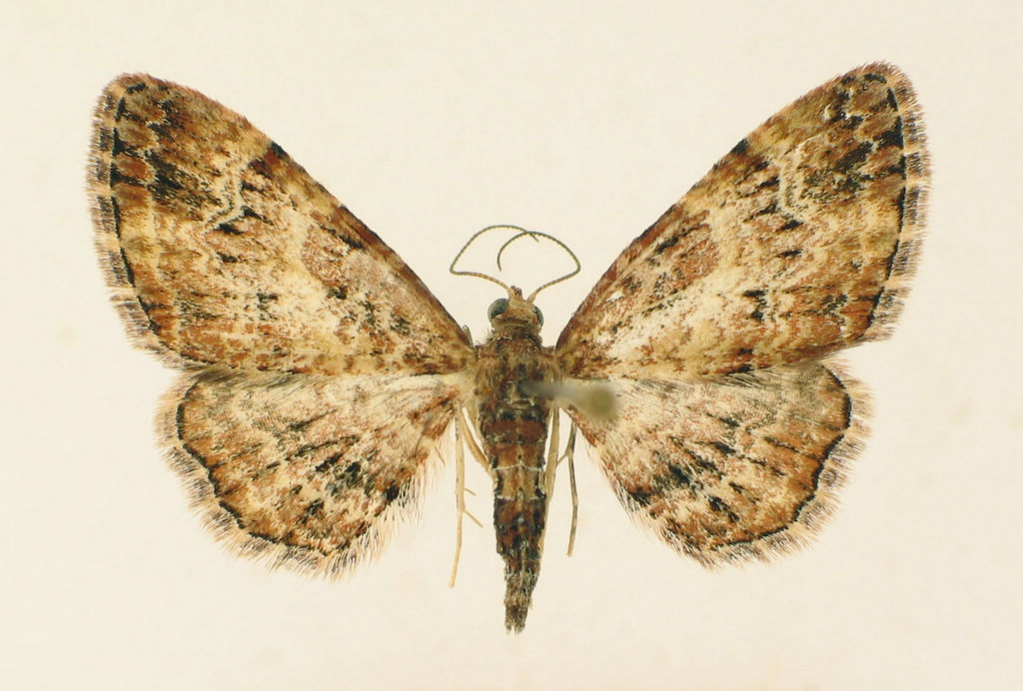
*Chloroclystis approximata*, female

**Figure 5.**
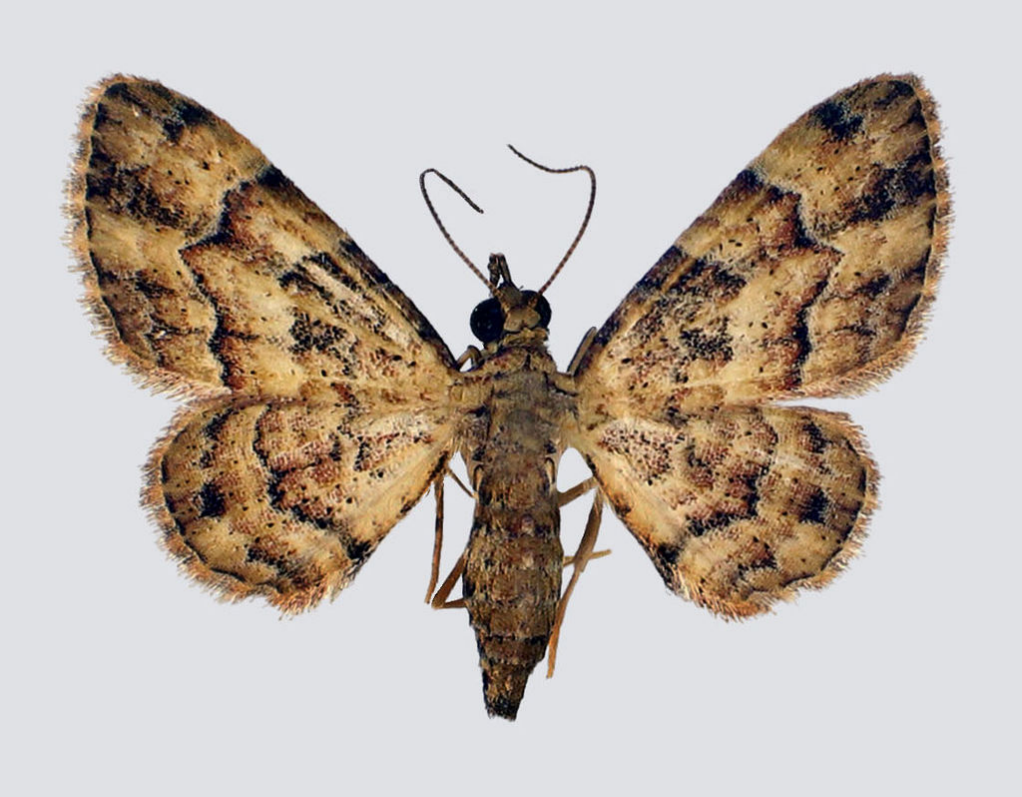
*Gymnoscelis lophopus*, female

**Figure 7.**
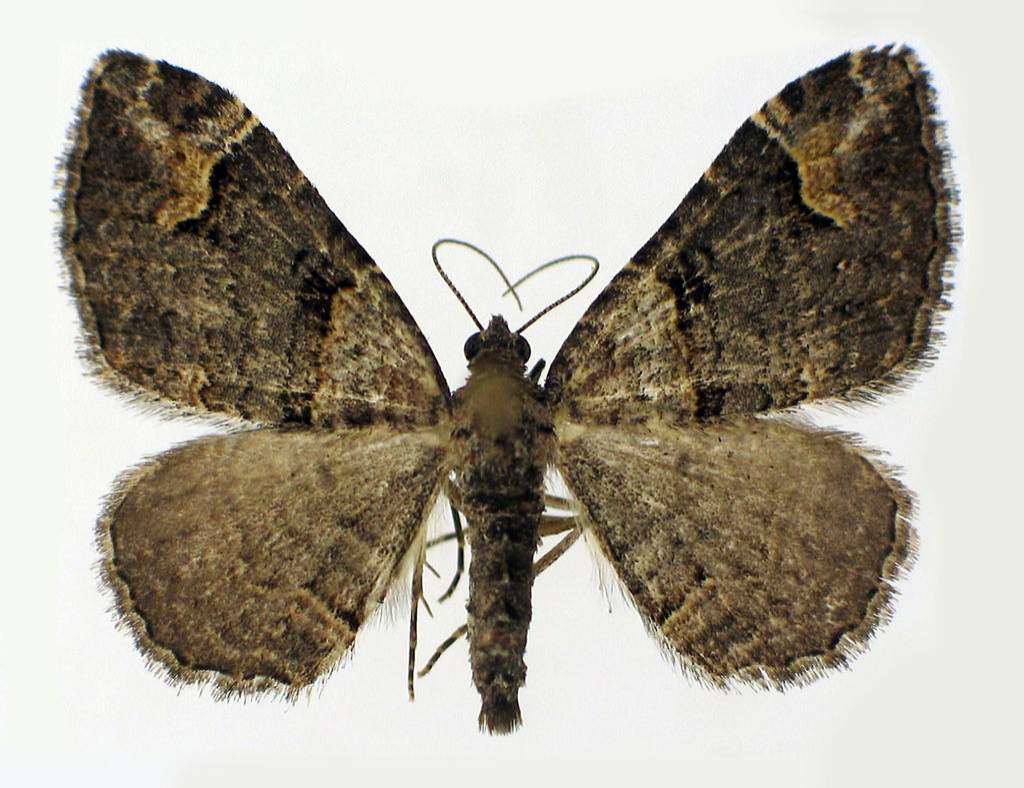
*Epyaxa sodaliata*, female

**Figure 9.**
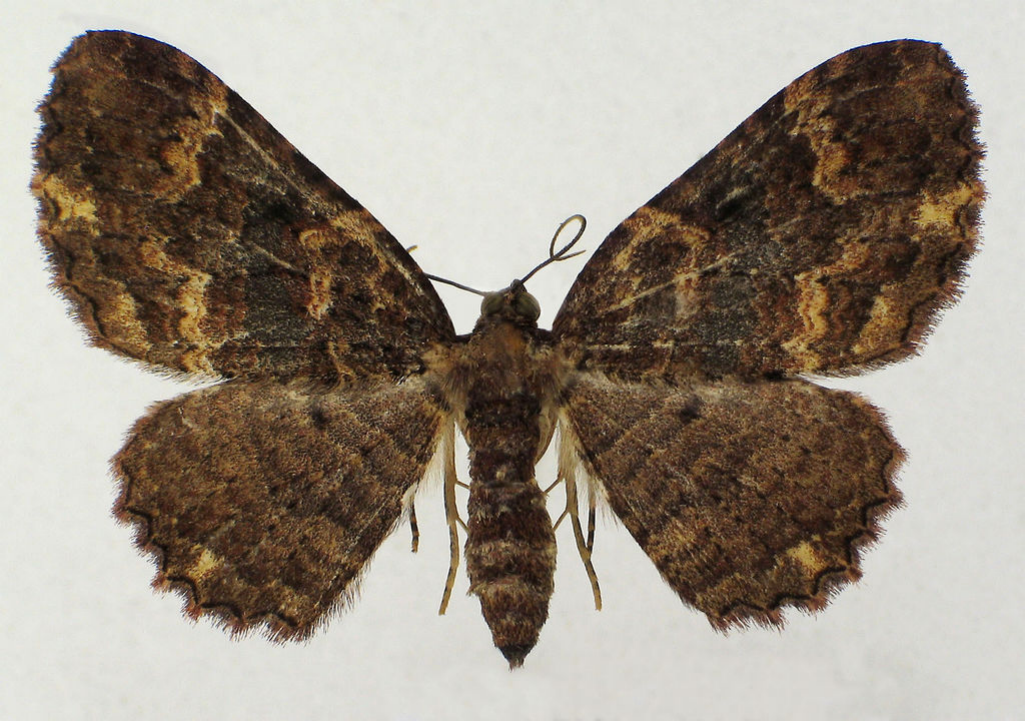
*Scotocyma albinotata*, female

**Figure 11.**
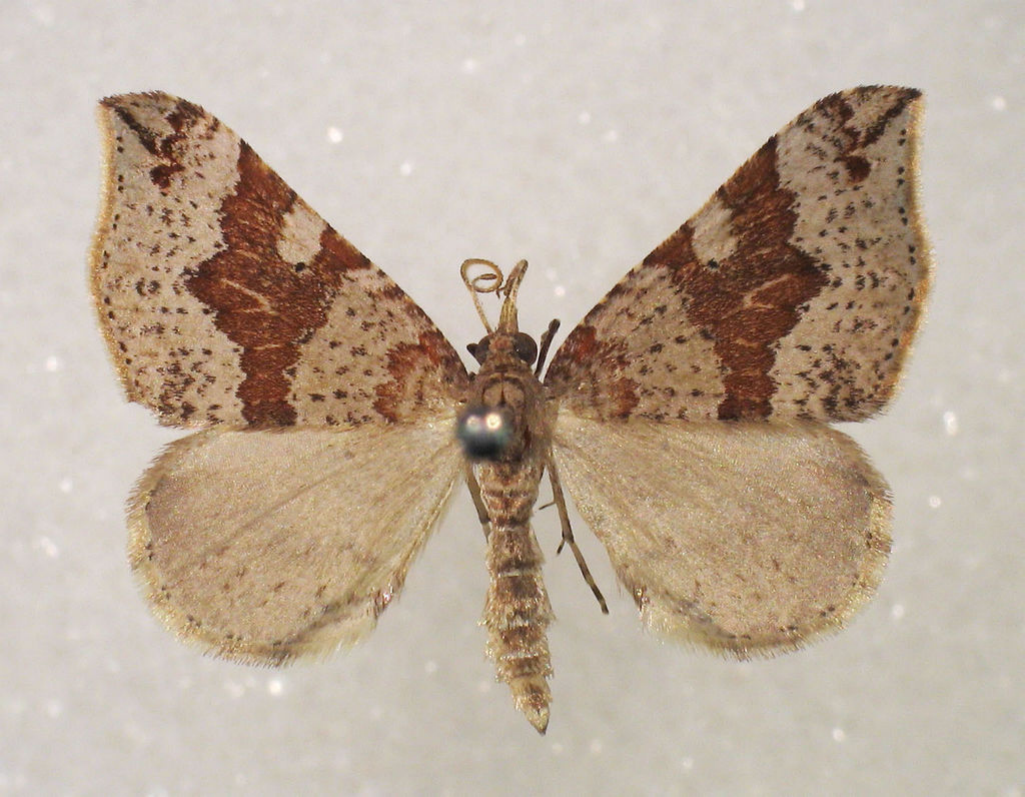
*Anachloris uncinata*, male

**Figure 13.**
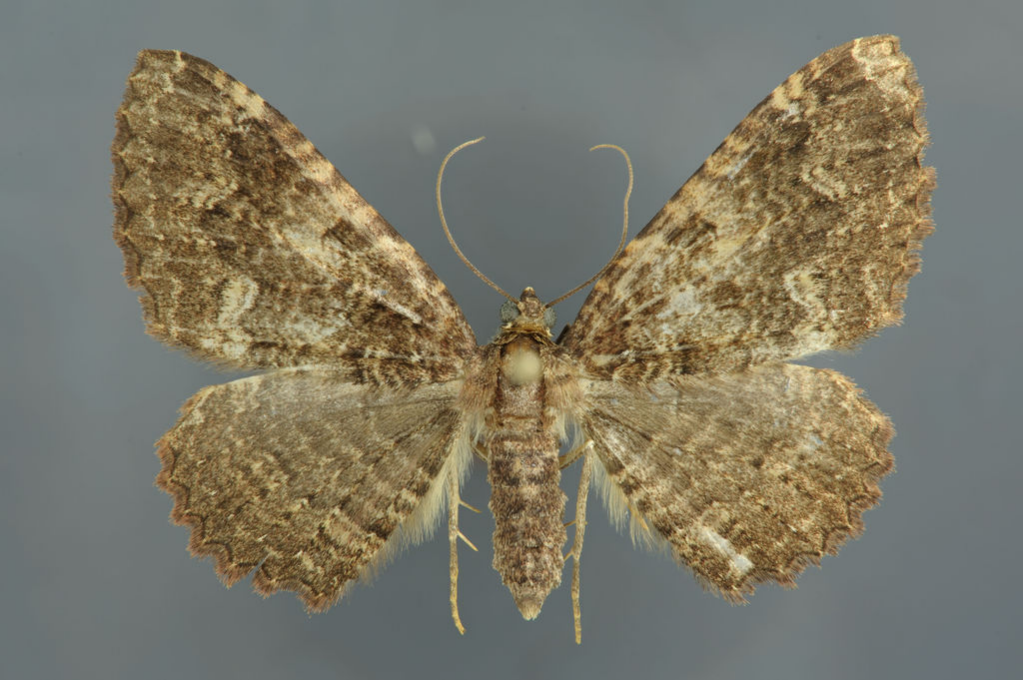
*Visiana incertata*, female

**Table 1.** Families of the larval food plants of Australian Larentiinae

**No**	**Food plant**	**Tribe**	**Species**
1	Araliaceae	Xanthorhoini	*Epyaxa subidaria*
2	Asteraceae	Eupitheciini	*"Chloroclystis" catastreptes*
2	Asteraceae	Eupitheciini	*"Chloroclystis" insigillata*
2	Asteraceae	Eupitheciini	*Microdes oriochares*
2	Asteraceae	Eupitheciini	*Phrissogonus laticostata*
2	Asteraceae	Eupitheciini	*Chrysolarentia vicissata*
2	Asteraceae	Unplaced to tribe	*"Chrysolarentia" squamulata*
3	Boraginaceae	Xanthorhoini	*Epyaxa sodaliata*
4	Caryophyllaceae	Xanthorhoini	*Chrysolarentia vicissata*
5	Chenopodiaceae	Xanthorhoini	*Chrysolarentia vicissata*
6	Dilleniaceae	Xanthorhoini	*Chrysolarentia vicissata*
6	Dilleniaceae	Unplaced to tribe	*Anachloris tofocolorata*
6	Dilleniaceae	Unplaced to tribe	*Anachloris uncinata*
6	Dilleniaceae	Unplaced to tribe	*Heterohasta conglobata*
7	Epacridaceae	Asthenini	*Poecilasthena pulchraria*
7	Epacridaceae	Unplaced to tribe	*"Chrysolarentia" leucophanes*
8	Ericaceae	Asthenini	*Poecilasthena pulchraria*
8	Ericaceae	Asthenini	*Phrissogonus laticostata*
8	Ericaceae	Unplaced to tribe	*"Chrysolarentia" severata*
9	Euphorbiaceae	Eupitheciini	*Bosara minima*
9	Euphorbiaceae	Eupitheciini	*"Chloroclystis" catastreptes*
9	Euphorbiaceae	Eupitheciini	*"Chloroclystis" insigillata*
10	Fabaceae	Eupitheciini	*"Chloroclystis" approximata*
10	Fabaceae	Eupitheciini	*"Chloroclystis" catastreptes*
10	Fabaceae	Eupitheciini	*"Chloroclystis" filata*
10	Fabaceae	Eupitheciini	*"Chloroclystis" insigillata*
10	Fabaceae	Eupitheciini	*Gymnoscelis lophopus*
10	Fabaceae	Eupitheciini	*Microdes squamulata*
10	Fabaceae	Eupitheciini	*Microdes villosata*
10	Fabaceae	Eupitheciini	*Pasiphila testulata*
10	Fabaceae	Eupitheciini	*Phrissogonus laticostata*
10	Fabaceae	Xanthorhoini	*Chrysolarentia vicissata*
10	Fabaceae	Xanthorhoini	*Epyaxa subidaria*
10	Fabaceae	Xanthorhoini	*Xanthorhoe vacuaria*
10	Fabaceae	Unplaced to tribe	*"Chrysolarentia" actinipha*
10	Fabaceae	Unplaced to tribe	*Melitulias* sp.
11	Gentianaceae	Xanthorhoini	*Chrysolarentia vicissata*
12	Geraniaceae	Asthenini	*Epicyme rubropunctaria*
12	Geraniaceae	Xanthorhoini	*Chrysolarentia decisaria*
13	Haloragaceae	Asthenini	*Epicyme rubropunctaria*
14	Lauraceae	Trichopterygini	*Sauris cirrhigera*
15	Lamiaceae	Xanthorhoini	*Chrysolarentia vicissata*
16	Lythraceae	Xanthorhoini	*Chrysolarentia vicissata*
17	Malvaceae	Xanthorhoini	*Chrysolarentia vicissata*
18	Meliaceae	Eupitheciini	*Symmimetis* sp.
18	Meliaceae	Trichopterygini	*Sauris malaca*
19	Moraceae	Unplaced to tribe	*Polyclysta hypogrammata*
20	Myrtaceae	Asthenini	*Poecilasthena balioloma*
20	Myrtaceae	Asthenini	*Poecilasthena ischnophrica*
20	Myrtaceae	Asthenini	*Poecilasthena pulchraria*
20	Myrtaceae	Asthenini	*Poecilasthena xylocyma*
20	Myrtaceae	Unplaced to tribe	*"Chrysolarentia" leucophanes*
21	Onagraceae	Unplaced to tribe	*Anachloris subochraria*
22	Piperaceae	Asthenini	*Poecilasthena pulchraria*
23	Pittosporaceae	Eupitheciini	*Gymnoscelis* sp.
24	Plantaginaceae	Eupitheciini	*"Chloroclystis" filata*
24	Plantaginaceae	Xanthorhoini	*Chrysolarentia insulsata*
24	Plantaginaceae	Xanthorhoini	*Chrysolarentia lucidulata*
24	Plantaginaceae	Xanthorhoini	*Chrysolarentia vicissata*
24	Plantaginaceae	Xanthorhoini	*Epyaxa subidaria*
25	Podocarpaceae	Trichopterygini	*Tympanota perophora*
26	Polygonaceae	Xanthorhoini	*Chrysolarentia vicissata*
27	Primulaceae	Eupitheciini	*Collix ghosha*
27	Primulaceae	Eupitheciini	*Epyaxa sodaliata*
28	Proteaceae	Eupitheciini	*"Chloroclystis" insigillata*
28	Proteaceae	Eupitheciini	*Gymnoscelis lophopus*
28	Proteaceae	Eupitheciini	*Gymnoscelis derogata*
29	Ranunculaceae	Eupitheciini	*"Chloroclystis" catastreptes*
29	Ranunculaceae	Eupitheciini	*"Chloroclystis" insigillata*
29	Ranunculaceae	Eupitheciini	*Phrissogonus laticostata*
29	Ranunculaceae	Xanthorhoini	*Chrysolarentia decisaria*
30	Rosaceae	Eupitheciini	*"Chloroclystis" approximata*
30	Rosaceae	Eupitheciini	*Pasiphila testulata*
30	Rosaceae	Eupitheciini	*Phrissogonus laticostata*
31	Rubiaceae	Eupitheciini	*Gymnoscelis delocyma*
31	Rubiaceae	Xanthorhoini	*Acodia* sp.
31	Rubiaceae	Xanthorhoini	*Austrocidaria* sp.
31	Rubiaceae	Xanthorhoini	*Scotocyma albinotata*
32	Santalaceae	Trichopterygini	*Sauris commoni*
33	Sapindaceae	Trichopterygini	*Sauris malaca*
34	Scrophulariaceae	Eupitheciini	*Chloroclystis s.l. sp.*
35	Urticaceae	Unplaced to tribe	*Visiana brujata*
35	Urticaceae	Unplaced to tribe	*Visiana incertata*
36	Verbenaceae	Eupitheciini	*Gymnoscelis lophopus*
